# Metabolite sensing and signaling in cell metabolism

**DOI:** 10.1038/s41392-018-0024-7

**Published:** 2018-11-09

**Authors:** Yi-Ping Wang, Qun-Ying Lei

**Affiliations:** Cancer Institute, Fudan University Cancer Hospital and Cancer Metabolism Laboratory, Institutes of Biomedical Sciences, Fudan University, 200032 Shanghai, People’s Republic of China

## Abstract

Metabolite sensing is one of the most fundamental biological processes. During evolution, multilayered mechanisms developed to sense fluctuations in a wide spectrum of metabolites, including nutrients, to coordinate cellular metabolism and biological networks. To date, AMPK and mTOR signaling are among the best-understood metabolite-sensing and signaling pathways. Here, we propose a sensor-transducer-effector model to describe known mechanisms of metabolite sensing and signaling. We define a metabolite sensor by its specificity, dynamicity, and functionality. We group the actions of metabolite sensing into three different modes: metabolite sensor-mediated signaling, metabolite-sensing module, and sensing by conjugating. With these modes of action, we provide a systematic view of how cells sense sugars, lipids, amino acids, and metabolic intermediates. In the future perspective, we suggest a systematic screen of metabolite-sensing macromolecules, high-throughput discovery of biomacromolecule-metabolite interactomes, and functional metabolomics to advance our knowledge of metabolite sensing and signaling. Most importantly, targeting metabolite sensing holds great promise in therapeutic intervention of metabolic diseases and in improving healthy aging.

## Metabolism: Three steps to sensing your environment

A simple, three-part model provides a systematic view of how cells sense sugars, lipids, amino acids and metabolic intermediates. Cells quickly and accurately perceive changes in intra- and extracellular molecules such as nutrients to respond to changing environments. Drawing on existing knowledge about AMPK and MTORC1 signaling, Yi-Ping Wang and Qun-Ying Lei at Fudan University in Shanghai propose a model in which three components: a sensor, transducer and effector enable metabolic sensing and signaling to proceed. The sensor detects the metabolite, and, through conjugation, conformational changes or protein–protein interactions, transmits this information to the transducer, which decides the appropriate response. The transducer then issues orders to effector proteins which coordinate the action. The future identification of novel metabolic sensors through systematic screening could lead to new therapeutic interventions for metabolic and age-related diseases.

At the very beginning of life, a key question followed the birth of the ancestor of cells: how to survive in the strange world? Metabolite sensing is one of the most fundamental biological processes implicated in cell proliferation, growth, differentiation, stress response, and ultimately, cell death.^1^ To interact with the environment and coordinate the biological network within, cells need a timely and accurate perception of the dynamic changes in intracellular and extracellular metabolites, particularly the concentration of nutrients. Functional metabolite-sensing machinery ensures the information exchange of a biological network and its habituating environment, assisting cells to thrive and survive in the long race of evolution.

The operation of a biological system requires extensive interaction between biological machinery (macromolecules) and the chemistry of its environment (different combinations and concentrations of chemicals/metabolites). In response to the changing environment, cells perform correspondingly to reorganize metabolic networks, modulate cell signaling, switch the cell cycle on and off etc.^2^ From unicellular organisms to evolutionarily more developed plants and mammals, nature has provided a multiple-layered and complicated toolbox for cells to sense and respond to a broad spectrum of metabolites.^1^ In bacteria cells, the lac operon efficiently confers cells with sugar (glucose and lactose)-sensing tactics.^3^ The lac operon elegantly employs sugar sensors and transcription factors to regulate the expression of metabolic enzymes and repurpose carbon metabolism in response to different carbon sources.^4^ In humans, in line with a multiplex biological buildup, cell metabolism is integrated into a highly interconnected biological network with a wider spectrum of metabolites and sophisticated metabolite-sensing mechanisms.

Metabolite sensing and signaling is the decision-making process of cells. Metabolite-sensing machinery allows cells to coordinate cellular metabolism with cell signaling and gene expression.^5^ After decades of metabolism research in cancer, immunology, and stem cell biology, we have never been closer to such an in-depth understanding of how cells coordinate their biology with the metabolic state. An enormous picture of metabolite sensing and signaling is unfolding in cell metabolism.

## A historical view of metabolite sensing and signaling

With the excellent efforts of early biochemists, metabolism research reached an unprecedented prosperity in the 1960s.^6^ Since then, a gigantic map of cellular metabolism has been depicted to define the origin and destiny of each nutrient and metabolite. With this map came the discouraging view that the uptake and utilization of nutrients were homeostatic, cell-autonomous, and disconnected from other biological events.^6^ To date, this classical metabolic network interconnecting numerous metabolites and enzymes remains independent chapters in biochemistry textbooks. Despite these frustrating facts, evidence supporting metabolite sensing has been accumulating in the early years of metabolism research.

### The availability of glucose and lactose controls gene expression in bacteria

The 1950s witnessed the discovery of the lactose operon.^7^ In this genetic paradigm, bacteria cells detect the availability of lactose and glucose by expressing two different proteins, lac repressor and catabolite activator protein (CAP), which directly bind to lactose and cyclic AMP (cAMP, an indicator of glucose), respectively.^7^ The metabolite-protein interaction would further affect the binding of the lac repressor and CAP with the lac operon, thereby fine-tuning the transcription/expression of enzymes and transporters involved in lactose catabolism.^4^ These findings suggest that cells actively sense the availability of carbon sources and modulate gene transcription accordingly to avoid wasteful protein synthesis.^8^

### Nutritional status regulates cell signaling

In the 1970s, high-glucose diets were observed to increase hepatic ATP levels and remodel nucleoli structure without altering the global phosphorylation of nuclear proteins.^9^ In contrast, under acute nutrient starvation, a ciliated protozoan (*Tetrahymena pyriformis*) showed pronounced phosphorylation of ribosomal protein S6.^10,11^ Another pioneering study demonstrated that short-term amino acid starvation of Landschutz tumor cells increased the phosphorylation of nuclear acidic proteins but not histones.^12^ These observations strongly suggest that protein phosphorylation is controlled by nutritional status, which means that nutrients potentially regulate signal transduction.

### Metabolites covalently modify proteins and modulate their function

In the 1960s, the NAD^+^ moiety was found to be incorporated into proteins.^13^ Shortly after, it was found that modification of elongation factor Ef-2 by ADP-ribose, which is dependent on NAD^+^, directly inhibits protein synthesis.^14,15^ Other evidence supporting metabolite-dependent covalent modification came from acetate. In 1970, HeLa cells were reported to take up acetate from the media and conjugate it onto histones,^16^ called histone acetylation. Although this observation was underappreciated at the moment of discovery, the significance of this modification is currently being intensively studied.^17^ Our group also found that acetate functions as an epigenetic metabolite to promote *do novo* lipid synthesis under hypoxia.^18^ Based on this evidence, it is reasonable to speculate that a wider species of metabolites are potentially modified onto macromolecules and exert regulatory functions.

## Nutrient/metabolite sensing: a ternary model

With decades of study, our knowledge of how metabolites modulate protein function, cell signaling, and gene expression has significantly expanded. Most notably, the discovery of AMPK signaling and mTORC1 signaling, both of which are master regulators of cell metabolism,^1^ further advanced our understanding of metabolite sensing and signaling.

### AMPK senses glucose and energy status

**Fig. 1:**
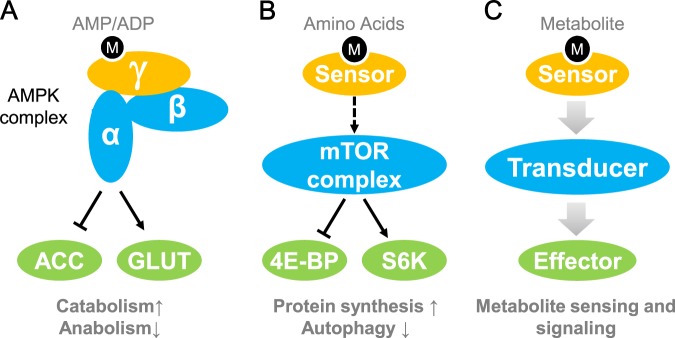
The model of metabolite sensing and signaling. **a** AMPK-mediated energy sensing and signaling. ACC, acetyl-CoA carboxylase; GLUT, glucose transporter. **b** mTOR-induced amino acid sensing and signaling. 4E-BP, eukaryotic translation initiation factor 4E-binding protein; S6K, ribosomal protein S6 kinase. **c** A working model composed of metabolite sensor (orange), signal transducer (blue), and effector (green). M indicates metabolite

AMPK was discovered in the 1970s as a 5′-AMP-activated protein kinase.^19^ AMPK is an evolutionarily conserved heterotrimer formed by a catalytic α subunit and two regulatory subunits (β and γ) (Fig. 1a). The γ subunit confers upon AMPK the ability to sense the AMP:ATP ratio. The γ subunit contains four cystathionine beta-synthase (CBS) domains, which are binding sites for AMP/ADP/ATP. In glucose shortage, when cells have an insufficient energy supply, the γ subunit of AMPK binds to AMP, thereby sensing the increased AMP/ATP ratio.^19^ Consequently, the AMP-bound γ subunit leads to a major conformational change in the AMPK heterotrimer complex, enabling the exposure of the catalytic pocket of the α subunit and activation of AMPK kinase.^20^ The α subunit further transmits this glucose shortage or energy crisis signal to its numerous downstream protein targets via phosphorylation events. For example, AMPK phosphorylates acetyl-CoA carboxylase 1 (ACC1) and sterol regulatory element-binding protein 1c (SREBP1c) to suppress lipid and cholesterol synthesis (Fig. 1a); AMPK phosphorylates ULK1 to enhance autophagy of damaged mitochondria and mitochondria biogenesis.^21^ AMPK also phosphorylates Rab-GAP protein TBC1D1 and promotes cell membrane translocation of GLUT4 to boost glucose uptake.^22^ These downstream proteins serve as effectors of AMPK signaling. Upon activation of AMPK, catabolism is enhanced to provide more energy, and anabolism is slowed down to avoid overdraft of the energy currency ATP (Fig. 1a). Consequently, cells maintain energy homeostasis with the assistance of AMPK, the glucose and energy sensor.

### mTOR signaling mediates amino acid sensing

Cells sense the availability of amino acids by using mTOR. Similar to AMPK, mTOR is also a protein kinase. The discovery of mTOR dates back to 1993 when the molecular target of rapamycin, a fungi-derived natural product with cell growth-suppressive activity, was identified.^23,24^ Shortly after, mTOR and its complex, named mTORC1 (mammalian target of rapamycin complex 1) were elucidated.^25^ Adequate building blocks for protein, i.e., amino acids, are a prerequisite for manufacturing protein. Amino acid availability positively regulates mTORC1 signaling (Fig. 1b). The action of mTORC1 employs extensive protein-protein interactions. Cells sense the presence of amino acids and switch Rag GTPase to its active conformation.^26^ Active Rag heterodimers further mediate mTORC1 activation through promoting the interaction between mTOR and Raptor.^27^ Activated mTOR further phosphorylates p70-S6 kinase 1 (S6K1) and 4E-BP1, the eukaryotic initiation factor 4E (eIF4E) binding protein 1^28^ (Fig. 1b). Once phosphorylated, S6K1 and 4E-BP1 activate protein translation initiation complexes and promote protein synthesis (Fig. 1b). In addition to S6K1 and 4E-BP1, mTORC1 exerts broad regulatory effects through phosphorylating other effector proteins.^29^

Notably, mTOR itself is not an amino acid sensor. mTORC1 reads the abundance of amino acids through complexing with specific sensor proteins (Fig. 1b). As protein synthesis is highly energy-demanding, the energy-sensing AMPK signaling pathway crosstalks with mTORC1 signaling.^30^

### A ternary model for metabolite sensing and signaling

Based on the action of AMPK signaling and mTORC1 signaling, we here propose a ternary model to illustrate metabolite sensing and signaling (Fig. 1c). In this model, three components, sensor, transducer, and effector, fulfill the mission of metabolite sensing and signaling. The sensor lies in the forefront of the metabolite-sensing model (Fig. 1c). With the help of metabolite sensors, cells efficiently integrate information on the fluctuations in different species of metabolites. In the AMPK complex, the γ subunit senses the AMP:ATP ratio, while sensors in mTORC1 signaling detect amino acid availability. Further, through conformational changes or protein-protein interactions, the sensor transmits the information to the transducer, similar to what the AMPK γ subunit does to the α subunit (the kinase subunit), and what amino acid sensors do to mTOR kinase. The transducer is in charge of the decision-making process within cells (Fig. 1c). The transducer is not necessarily a protein but may be a signaling pathway or a set of signaling pathways. The transducer compiles the information input transferred from sensors on metabolite abundance, nutritional status, and energy status. After a series of signaling events and complex formation/dissociation, the transducer gives orders to effector proteins, which are executors of the biological output of the metabolite signal (Fig. 1c). For AMPK and mTORC1 signaling, the effectors are downstream targets of the AMPK α subunit and mTOR kinase, respectively. We would use this sensor-transducer-effector model in this review to explain the current understanding of metabolite sensing and signaling.

## Metabolite sensor: where everything begins

In a metabolite-sensing and signaling pathway, the metabolite sensor lies at the interface of the environment and biological networks. The sensor directly perceives metabolite information in the environment, and then the sensor protein transforms the chemical signals into cell signaling events in collaboration with a transducer. The transducer is generally a signaling pathway(s). These signaling pathways converge or diverge and fulfill the decision-making process. Effector molecules receive the signal from the transducer and mediate the responses to the metabolite signal, mostly coordinating metabolic activity with the nutritional or stress status of cells. In this ternary model, the sensor is undoubtedly the most important part of metabolite sensing/signaling.

The sensor is a term that originates from engineering.^31^ In the broadest definition, the sensor is defined as a device or a module that detects a stimulus or changes in its environment and transmits the information to another device.^31^ A metabolite sensor can be defined as a biological molecule that detects the changes/presence of a specific metabolite and transmits the information of metabolite abundance into biological networks. A metabolite sensor is a biological macromolecule, that is, protein, RNA or even DNA, that functions at the interface of metabolite and signaling pathways. A metabolite sensor binds directly to the metabolite and induces changes in its downstream protein. Metabolite sensors have a well-defined metabolite-binding domain and stably exist in the cells to read the abundance information of target metabolites. Metabolite sensing is located at the interface of a biological network and its environment, intracellularly and extracellularly. Based on the current understanding of metabolite sensing, we propose three criteria that define a sensor.

1. **Specificity**: the sensor recognizes and binds to a metabolite using a structurally recognizable domain. The binding of the sensor to the metabolite is highly specific to ensure the accuracy of metabolite sensing.^32^
2. **Dynamicity**: the binding of the sensor to the metabolite is reversible, meaning that the metabolite signal can be switched on and off. The dissociation constant of this binding lies within the physiological range of the metabolite, which allows the sensor to sense the fluctuation of the metabolite. In this regard, the identity of a metabolite sensor can be validated by a competitive metabolite-binding assay;^33,34^ that is, free metabolite (usually isotope-labeled) can compete with the sensor-bound metabolite.
3. **Functionality**: in cells, the binding and dissociation of the metabolite modulate the activity/function of the sensor through modulating protein conformation or protein–protein interaction. Specifically, the function of the sensor is to transform the chemical signal of a metabolite (concentration) into a biological signal that is communicable within biological networks. The sensor plays the role of environment translator for cells.

## Metabolite sensing and signaling: modes of action

Cells adopt different mechanisms to transmit changes in metabolites into their biological network. In general, cells employ different modes of metabolite-sensing mechanisms. Here, we group different metabolite-sensing modes into three categories: metabolite sensor-induced signaling, metabolite-sensing module, and sensing by conjugation, to review recent advances in metabolite sensing. Notably, metabolite sensing may employ multiple modes of mechanisms.

### Metabolite sensor-mediated signaling

In this category, a defined metabolite sensor physically interacts with the metabolite and signals to downstream proteins.

#### AMPK-mediated glucose and FBP sensing

**Fig. 2:**
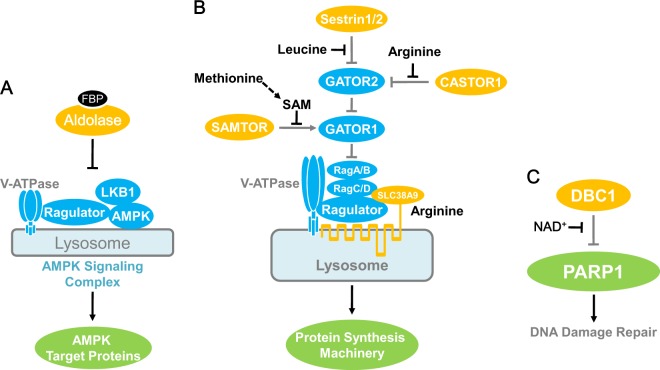
Metabolite sensor-mediated signaling. **a** Aldolase senses FBP and signals to AMPK. FBP, fructose 1,6-bisphosphate; V-ATPase, vacuolar-type H^+^-ATPase; Ragulator, protein complex that interacts with the Rag GTPases; LKB1, liver kinase B1. **b** Sestrin, CASTOR1, and SAMTOR mediate the sensing and signaling of leucine, arginine, and SAM, respectively. GATOR1/2, GATOR complex 1/2; Rag, Rag GTPases; SLC38A9, an amino acid transporter, also functions as a putative arginine sensor. **c** DBC1 signals NAD^+^ level to PARP1

The classical model of AMPK-dependent glucose sensing employs the γ subunit as the sensor for the AMP:ATP ratio. Recently, AMPK was found to use an AMP/ATP-independent mechanism to induce glucose sensing. Upon glucose depletion, an intermediate of glycolysis, fructose-1,6-biphosphate (FBP), is dramatically decreased. A glycolytic enzyme aldolase functions as a glucose sensor by sensing FBP.^35^ FBP-unbound aldolase promotes the association of AMPK with v-ATPase, ragulator, axin, and liver kinase B1.^35^ By modulating the complex formation, aldolase signals glucose availability to AMPK (Fig. 2a).

#### GPCRs as sensors for TCA cycle metabolites

Transmembrane proteins may serve as metabolite sensors or metabolite receptors. A G-protein-coupled receptor (GPCR), GPR91, previously thought to be an orphan receptor, was demonstrated to function as a succinate receptor. Succinate activates GPR91 at a half-maximal response (EC50) of 25–56 μM in vitro. Another GPCR, GPR99, functions as an α-ketoglutarate receptor with an EC50 of approximately 32–69 μM. A partial three-dimensional simulation suggests that succinate and α-ketoglutarate bind to the basic central cavity of GPR91 and GPR99, respectively. This observation is in agreement with the finding that the dicarboxylate group of the metabolite ligand is necessary for GPCR activation. Moreover, GPR91 and GPR99 showed tissue-specific expression. While both GPCRs were expressed in the cortical region of mouse kidneys, GPR91 was mainly expressed in proximal tubules and GPR99 in distal tubules. Functional experiments further demonstrated that GPR91 signaled succinate to downstream signaling pathways and mediated hypertensive effects through activating the renin-angiotensin system.^36^

#### mTORC1-mediated amino acid sensing

mTOR kinase cannot function as an amino acid sensor itself. With the help of arginine sensor proteins, the arginine level controls mTOR activity. The arginine sensor controls mTOR activity by modulating mTOR complex formation. Two different proteins, SLC38A9 and CASTOR1, have been shown to be putative arginine sensors (Fig. 2b). SLC38A9, which is a lysosomal arginine transporter, functions as a potential arginine sensor. Transportation of arginine by SLC38A9 has a high Km. Moreover, SLC38A9 interacts with the Rag GTPases and ragulator in an arginine-dependent manner. In the presence of abundant arginine, SLC38A9 promotes the formation of an active mTOR complex and signals arginine sufficiency to mTORC1.^37^ When cells are in a shortage of arginine, CASTOR1 functions as an arginine sensor to inhibit mTORC1 (Fig. 2b). Arginine directly binds to the ACT domain of CASTOR1 and disrupts the association of CASTOR1 with GATOR2 (a complex of mTORC1 regulatory factors), thereby blocking the inhibitory effect of CASTOR1 and activating mTORC1. Arginine physically interacts with CASTOR1 with a dissociation constant (Kd) at 24.2–34.8 μM.^33^ The leucine sensing of mTORC1 is performed in a similar fashion. Sestrin2 serves as leucine sensor for mTORC1 (Fig. 2b). Leucine-bound Sestrin2 is released from GATOR2, leading to mTORC1 complex activation. The Kd of leucine, when bound to Sestrin2, is approximately 20 μM. Structural studies demonstrate that Sestrin2 has a leucine-binding pocket. Mutations in this pocket alter the affinity towards leucine.^32,34^ In addition, mTORC1 employs SAMTOR as a SAM/methionine sensor (Fig. 2b). SAM binds to SAMTOR with a Kd at approximately 7 μM and disrupts its interaction with GATOR1, the GTPase-activating protein for Rag subunits A/B.^38^ The cellular SAM level is closely linked with methionine availability. Methionine starvation reduces the SAM level and consequently suppresses mTORC1 signaling by enhancing SAMTOR-GATOR1 binding.^38^

#### DBC1-dependent NAD^+^ sensing

One of the hallmarks of aging is the accumulation of DNA damage. NAD^+^ supplementation has been reported to show rejuvenating effects on aged animals.^39^ This may be attributed to a DBC1 (deleted in breast cancer 1)-dependent NAD^+^-sensing pathway (Fig. 2c). In a homology-based structural modeling, NAD^+^, but not other riboside nucleotides, specifically binds to the NHD domain (nudix homology domain) of DBC1. Within physiological ranges, NAD^+^ disrupts the interaction of DBC1 and PARP1 in a dose-dependent manner (Fig. 2c). Consequently, NAD^+^-bound DBC1 fails to bind to and inhibit PARP1, a DNA repair enzyme. In aged animals, the level of NAD^+^ is usually decreased, resulting in enhanced DBC1-PARP1 interaction and compromised DNA damage response^39^ (Fig. 2c). Collectively, DBC1 functions as an NAD^+^ sensor and signals NAD^+^ sufficiency to DNA damage repair.^39^

NAD^+^ sensing can also be carried out through modulating the acetylation status of proteins, as NAD^+^ is required for SIRT-catalyzed deacetylation reactions.^40,41^ Of note, allosteric modulation of metabolic enzymes belongs to metabolite sensing within metabolic networks. For example, the allosteric activator of PKM2, FBP, serves as an indicator of glycolysis.^42^ PKM2 senses FBP availability and undergoes a dimer-tetramer transition to promote glycolysis.^42,43^

Metabolite transporters hold great promise to function as sensors. This concept is at least in part supported by the contribution of lysosomal transporter SLC38A9 to arginine sensing.^33^ Of note, various transporters, including SLC7A11 (cystine/glutamate transporter), ASCT2/SN2 (glutamine transporter), and MCT1/4 (lactate transporter), potentially mediate the signaling of corresponding metabolites.^44^ These transporters potentially function in both delivering metabolites and mediating metabolite signaling.

### Metabolite-sensing module

A metabolite-sensing module is composed of more than one molecule and lacks a structurally conserved metabolite-binding site. Molecules in this module have to act in concert to fulfill the metabolite-sensing function.

#### NDRG3-VHL module mediates lactate/hypoxia sensing

**Fig. 3:**
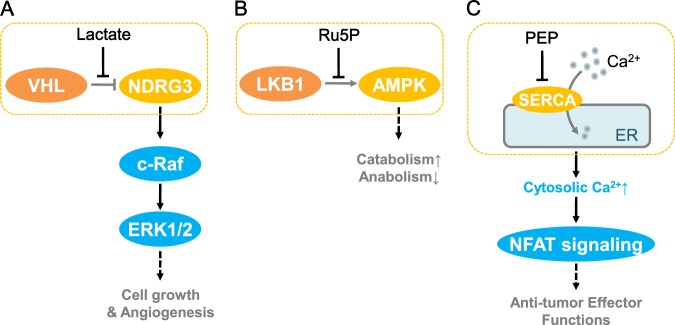
Metabolite-sensing module mediates metabolite signaling. **a** NDRG3 mediates lactate sensing. VHL, Von Hippel–Lindau tumor suppressor; c-Raf, RAF proto-oncogene serine/threonine-protein kinase; ERK, extracellular signal-regulated kinase. **b** LKB1-AMPK complex mediates Ru5P sensing. Ru5P, ribulose -5-phosphate. **c** SERCA and ER mediate PEP sensing and anti-tumor signaling in T cells. PEP, phosphoenolpyruvate; SERCA, sarco/endoplasmic reticulum Ca-ATPase; NFAT, Nuclear factor of activated T cells. The yellow dotted box indicates the metabolite-sensing module

Due to enhanced glycolysis, cells accumulate lactate in the face of oxygen insufficiency. When oxygen is scarce, HIF prolyl-hydroxylases (PHD)-mediated hydroxylation of HIF, which is oxygen dependent, is inhibited.^45^ As a result, HIF is stabilized, and the hypoxia response is induced. However, cells adopt a HIF-independent mechanism to sense the accumulation of lactate.^46^ Upon hypoxia, lactate is sensed by NDRG3, which leads to the disruption of the NDRG3-VHL complex and NDRG3 stabilization. Further, NDRG3 triggers Raf-ERK signaling and mediates lactate signaling by promoting angiogenesis and cell proliferation (Fig. 3a).

#### LKB1-AMPK module mediates Ru5P sensing

Biosynthesis requires both energy and building blocks. As an energy sensor, AMPK also functions as a checkpoint for the sufficiency of biosynthetic precursors. The oxidative branch of the pentose phosphate pathway provides both ribose and NADPH for manufacturing nucleotides and lipids.^47,48^ AMPK senses the activity of oxidative PPP by an LKB1-AMPK sensing module (Fig. 3b). In this module, ribulose-5-phosphate, an intermediate in oxidative PPP, disrupts the interaction of AMPK with its activating kinase LKB1.^49^ Thus, the LKB1-AMPK module senses the increase in ribulose 5-phosphate (Ru5P), i.e., the ready-to-go signal for the biosynthesis of lipids and nucleotides (Fig. 3b). Consequently, AMPK activity is decreased, and lipogenesis is correspondingly activated.

#### The SERCA-ER-sensing module functions as a metabolic checkpoint in T cells

Tumor-infiltrating T cells consume glucose as a carbon source for their antitumor function. In a glucose-restricted tumor microenvironment, T cells have a low level of aerobic glycolysis. The glycolytic state of T cells is sensed by a SERCA-ER module, which detects the level of phosphoenolpyruvate (PEP), an intermediate in glycolysis (Fig. 3c). SERCA (Sarco/ER Ca^2+^-ATPase) is an ER membrane-bound calcium transporter that mediates the Ca^2+^ influx of ER. The SERCA-ER module senses PEP, and with the potential help of unknown proteins, mediates the cysteine oxidation and inhibition of SERCA.^50^ This further causes increased cytosolic Ca^2+^ and activates NAFT signaling to boost tumor immunosurveillance (Fig. 3c). Upon glucose deprivation, SERCA-ER senses the decrease in PEP, and the function of tumor-reactive T cells is compromised.^50^

### Sensing by conjugating

**Fig. 4:**
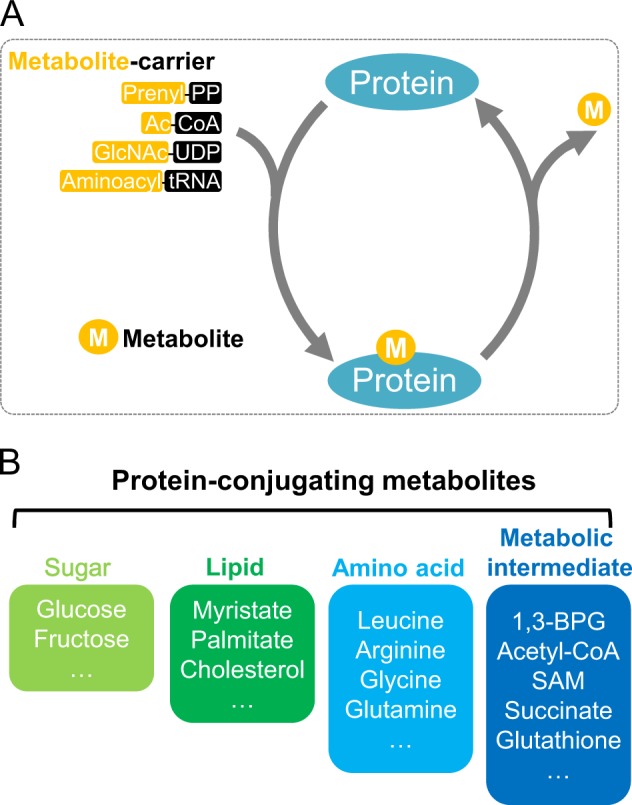
Metabolite sensing by conjugating. **a** Scheme of modification of proteins by metabolites. Prenyl-PP, prenyl diphosphate; Ac-CoA, acetyl-CoA; GlcNAc-UDP, uridine diphosphate N-acetylglucosamine. **b** Proteins are conjugated with sugar, lipid, amino acid and metabolic intermediates. The boxes indicate which metabolites are covalently linked to proteins

Metabolites are conjugated to proteins or nucleotides, causing functional impacts on the modified molecules. To date, a wide spectrum of metabolites has been shown to be covalently linked to proteins and modulate their activity^51^ (Fig. 4a). As covalent linkage of metabolites to proteins occurs mostly in an enzyme-dependent manner, the modification of proteins is potentially linked to the abundance of the metabolite.^52,53^ As such, metabolite conjugation serves as a key mechanism of metabolite sensing. During metabolite conjugation, a carrier moiety is frequently employed to facilitate the enzymatic transfer of the metabolite (Fig. 4a).

#### Sugar modification

Sugar can be attached to proteins through glycation (non-enzymatic) or glycosylation (ATP-dependent modification). In glycation reactions, glucose or fructose is covalently bound to protein and modifies its function (Fig. 4b). In glycosylation reactions, the sugar group is linked to a protein with the help of enzymes, such as O-linked GlcNAc transferase (OGT).^54^ OGT is involved in cellular metabolism and epigenetics through mediating glucose sensing.^54,55^ In response to hypoxia, OGT promotes the glycosylation of G6PD, the rate-limiting enzyme in PPP. Glycosylation of G6PD further increases its activity and promotes the anabolism of nucleotides and lipids, thereby supporting cancer proliferation.^56^ OGT also signals glucose availability to TET3 by mediating its glycosylation. The DNA hydroxylating enzyme TET3 is hypo-glycosylated when cells are cultured in low glucose conditions, which is coupled with enhanced nuclear localization of TET3. The epigenetic effect of this regulation remains to be defined.^57^

#### Lipid modification

As an insoluble metabolite, lipids are attached to proteins to modulate the activity and mostly subcellular localization of proteins.^58^ Lipid attachment of proteins involves a variety of lipid species, such as myristate, palmitate, and cholesterol^59^ (Fig. 4b). The synthesis of lipids is coupled with the activity of the modified protein. As a lipidated protein, RHO GTPases serve as targets for lipid sensing.^60,61^ Geranylgeranyl-PP is a key intermediate representing the activity of mevalonate metabolism. Geranylgeranyl-PP also serves as a geranylgeranyl donor for the lipid modification of RHO GTPases. When mevalonate metabolism is active, RHO GTPase is geranylgeranylated.^60,61^ Geranylgeranylated RHO GTPase shows enhanced membrane localization, where it promotes YAP/TAZ activity to accelerate cell growth.^60,61^ Together, geranylgeranylation modification serves as a sensing mechanism to connect mevalonate metabolism with YAP/TAZ signaling.

#### Modification of protein by amino acids

Amino acids can be attached to proteins as post-translational modifications (Fig. 4b). The first knowledge of amino acid conjugation came in 1974, when brain tubulin was reported to be tyrosinated.^62^ Tubulin tyrosine ligase (TTL) and tubulin carboxypeptidase (TCP) tyrosinate and detyrosinate the C-terminus of alpha-tubulin by catalyzing the formation and breakage of the peptide bond, respectively.^63^ In the 1990s, glutamate and glycine joined the company. Glutamate and glycine were found to be modified on the γ-carboxy group of glutamate residues in tubulins by forming a peptide-like bond.^64,65^ Interestingly, glutamate and glycine can be polyconjugated onto proteins in a chain-like fashion. The tubulin tyrosine ligase-like (TTLL) family of proteins attaches glutamate and glycine to proteins,^66^ while certain carboxypeptidases and metallopeptidases remove these modifications.^67,68^ In the tyrosination, glutamylation, and glycylation reactions, these three amino acids are attached to proteins as free metabolites. The amino acid ligase and peptidases write and erase these modification markers without the help of a metabolite carrier. As the cytoskeleton intensively crosstalks with cell metabolism and cell signaling, the abundance of amino acids can be reasonably speculated to modulate amino acid modification of tubulin and consequently nutrient transportation and cell movement.

Amino acids can also be covalently linked to protein in a manner called aminoacylation. In 1965, it was found that with a cell-free system deficient for protein synthesis, radioactive amino acids were still incorporated into protein in a tRNA-dependent manner.^69^ This observation led to the discovery not of unconventional peptide elongation but of protein aminoacylation. Subsequent studies documented that this tRNA-dependent aminoacylation was specifically restricted to arginine, leucine, and phenylalanine.^70^ In eukaryotic cells, arginine was thought to be the only amino acid that was added to proteins.^70^ Extensive biochemical studies further demonstrated three features of protein aminoacylation: (a) similar to protein translation, protein aminoacylation employs aminoacyl-tRNA to carry out the transfer of amino acid. tRNA serves as the metabolite carrier in this type of modification; (b) aminoacylation is dependent on the corresponding aminoacyltRNA synthetase, which determines the specificity of aminoacylation; (c) aminoacylation is highly variable in covalent bond formation and in substrate specificity. Arginine can form an amide bond with the carboxyl group of glutamate protruding from the peptide chain or with the N-terminal-exposed amino group of acceptor protein.^71^ Arginine, leucine, and phenylalanine can be incorporated into the N-terminus or an internal position of the peptide.^72^ A mass spectrometry study in 2007 identified 43 argininylated proteins that were involved in cytoskeleton and metabolism.^73^ Most of these 43 proteins were modified in internal residues of the protein. This fashion of argininylation was attributed to the attachment of arginine to the side chains of lysine, serine, threonine, and some other amino acids.^73^ Together, these observations suggest that protein aminoacylation has a versatile chemistry, and more importantly, aminoacylation of proteins regulates protein function in a sophisticated manner. Recently, an illuminating finding further demonstrated that, in addition to arginine, the other 19 proteogenic amino acids were readily attached to proteins in human cells.^74^ Each aminoacyl-tRNA synthetase transferred the cognate amino acid on to the ɛ-amine of lysines in modified proteins. Notably, Ras-related GTP-binding protein A/B (RagA/B), a key component in mTORC1 signaling, was found to be leucylated. Leucylation of RagA/B signaled leucine sufficiency to mTOR kinase and modulated amino acid metabolism.^74^ Collectively, amino acid modification of proteins serves as an important mechanism of transmitting amino acid signal into biological networks.

#### Protein modification by metabolic intermediates

When discussing protein-modifying metabolites, metabolic intermediates cannot be underestimated (Fig. 4b). 1,3-BPG, the intermediate of glycolysis, is covalently attached to lysine side chains and generates 3-phosphoglyceryl-lysine.^75^ Glycolytic enzymes are among the major acceptors of this modification. 1,3-BPG transmits high glucose signals to glycolytic enzymes and suppresses their activity. When cells are exposed to high glucose, they sense 1,3-BPG level to mediate negative feedback on glycolysis.^75^ TCA cycle metabolites can also be covalently added onto proteins. Succination of lysine residues signals succinate to its target proteins and coordinates TCA cycle,^76^ mitochondria respiration,^77^ and lysosomal function.^78^

Another group of metabolites that is attached to proteins is the small-molecule organic acids (Fig. 4b). Notably, histone is a key acceptor of these organic acids. Methyl groups can be attached to the lysine and arginine residues in histone and regulate autophagic process.^79^ Moreover, the cellular level of methyl donor SAM is modulated by methionine metabolism. Methionine cycle activity is signaled by SAM to specific histone methylation events, thereby coupling one-carbon metabolism with gene regulation.^80^ Histone is also modified by acetyl groups at its lysine residues. Acetyl-CoA, which comes from glucose and acetate, is the acetyl donor for acetylation.^18,81–84^ Lysine acetylation is a prevalent modification within cells. Protein acetylation plays a key role in transmitting carbon source availability to metabolism, signaling, and epigenetics.^85,86^ Additionally, histone is modified by a variety of organic acids,^51^ of which the potential sensing/signaling function remains to be explored.

Interestingly, glutathione, a metabolite involved in redox homeostasis, is added on to cysteine residues of proteins under oxidative stress (Fig. 4b). Glutathionylation serves as a key redox signaling mechanism and mediates oxidative stress responses.^87^ Other metabolic intermediates, such as NAD^+^, can also be conjugated to proteins and mediate metabolite signaling.^88^ Of note, NAD^+^ also serves as a substrate for SIRT family deacetylases. SIRT is also considered as an energy sensor by decreasing the acetylation/aminoacylation/succinylation of proteins.^89^

## Future perspectives

Metabolite sensing is a fast-evolving field. Some key questions have been pursued for a long time. How do cells rewire metabolic networks when supplied with different nutrients? How do cells read and transmit environmental metabolic intel (nutrients or metabolic stress) to intracellular biological machinery? To answer these questions, we need to put more effort into studying the metabolite-sensing and signaling machinery.

### Metabolite sensor as a broader concept

Currently known metabolite sensors are all proteins, either enzymes or metabolite-bound proteins. However, RNA and DNA are also macromolecules that play a key regulatory role in cell metabolism, signaling, and epigenetics. The functional region of RNA for metabolite binding is termed the riboswitch.^90^ Although most known riboswitches are identified in bacteria, mammalian cells may have retained this regulatory mechanism during evolution. Riboswitches bind to multiple metabolites, such as nucleotide derivatives, amino acids, metal ions, purine, and SAM/SAH.^91^ If an RNA molecule does serve as a metabolite sensor, it may transmit the signal through conformational changes that affect RNA–RNA interaction or RNA–protein interaction. More work in RNA biology and RNA metabolism will help to determine if the concept of the sensor can be extended.

### Systemic screening of metabolite sensors

Metabolites are known for the complexity and diversity of their chemical properties, which hinder high-throughput screening of metabolite sensors. A complementary approach is to identify metabolite-bound proteins or candidates for metabolite sensors. Recently, this strategy has seen success in identifying metabolite-bound soluble protein and lipid-bound protein using a proteomic approach.^92,93^ As the sensor translates the abundance of a metabolite into downstream signals, whether a metabolite-bound protein functions as a *bona fide* sensor can be individually tested under metabolite-saturated or metabolite-depleted conditions. A possible experimental scheme is to interrogate whether these potent sensors modulate the activity of their downstream targets in response to metabolite availability. The spectrum of metabolites that is being sensed, and the mechanism of how cells sense different metabolites, remain poorly understood. A deep understanding of the metabolite-protein interactome, metabolite-RNA interactome, or even metabolite-macromolecule interactome will pave the way for metabolite-sensing research.

### Refining the biological function of metabolite sensing

Metabolic behavior varies across different cell types. Metabolic activity is intimately linked with differentiation state and even malignancy.^94^ Within a tissue, metabolite sensing and signaling may be cell-context dependent. More specifically, different sensing mechanisms may be employed for the same metabolite in two different cells. In addition, metabolites, similar to the enzymes that metabolize them, show compartmentalized distribution within cells.^95^ Thus, different organelles may utilize distinctive mechanisms of metabolite sensing and mediate inter-organelle crosstalk of metabolite signaling events. To better understand how cells metabolically act in concert, we must profile the metabolite-sensing pathways at subcellular and cellular levels. A functional metabolomics approach would help nail down the biological significance of metabolite sensing.^96^ Single-cell proteomics and metabolomics, although technically challenging, would definitely advance our knowledge of metabolite sensing and signaling towards the single-cell level.

### Targeted intervention of metabolite sensing

The fundamental role of metabolite sensing and signaling renders metabolite sensors as therapeutic targets in various metabolic diseases. As a master regulator of cell proliferation, mTORC1 signaling is activated by multiple oncogenic mutations. mTORC1 is thought to be upregulated in approximately 70% of all human cancers.^97^ To date, two generations of mTOR inhibitors have been developed that demonstrate promising tumor-inhibitory effects in preclinical studies. Clinical application of mTOR inhibitors has seen success in treating advanced renal cell carcinoma, neuroendocrine tumors, and HER2-positive breast cancer.^97^ In addition, AMPK is a vital therapeutic target in treating obesity, insulin resistance, and non-alcoholic fatty liver disease.^98^ Interestingly, the intervention of NAD^+^ sensing has beneficial effects in aged animals. Restoration of cellular NAD^+^ improves the health of diseased animals and prolongs the lifespan of aged mice.^99^ The NAD^+^ sensor sirtuin is an attractive target for coping with inflammatory diseases and neurodegeneration.^100^ Collectively, the discovery of new metabolite sensors would open a wide range of opportunities to therapeutically target metabolite sensing and signaling.
